# Characterisation of Primary Human Hippocampal Astrocyte Cell Culture Following Exposure to Hypoxia

**DOI:** 10.21315/mjms2023.30.1.8

**Published:** 2023-02-28

**Authors:** Nurul Atikah Nor Nazli, Sangu Muthuraju, Farizan Ahmad, Abdul Aziz Mohamed Yusoff, Hasnan Jaafar, Shaharum Shamsuddin, Jafri Malin Abdullah

**Affiliations:** 1 Department of Neurosciences, School of Medical Sciences, Universiti Sains Malaysia, Kelantan, Malaysia; 2 Brain and Behaviour Cluster, School of Medical Sciences, Universiti Sains Malaysia, Kelantan, Malaysia; 3 Department of Pathology, School of Medical Sciences, Universiti Sains Malaysia, Kelantan, Malaysia; 4 Department of Biomedicine, School of Health Sciences, Universiti Sains Malaysia, Kelantan, Malaysia

**Keywords:** hypoxia, human hippocampal astrocytes, oxygen percentage, cell viability, morphological changes, annexin V-fluorescein isothiocyanate staining, glial fibrillary acidic protein marker, HIF-1α, glyceraldehyde 3-phosphate dehydrogenase, B-cell lymphoma 2

## Abstract

**Background:**

The present study aimed to understand the characterisation of human hippocampal astrocyte following hypoxia exposure. Based on the preliminary screening, 15 min was chosen as the time point and the cells were exposed to different oxygen percentages.

**Methods:**

The Trypan blue viability assay used to examine cell death. Immunofluorescence assay, glial fibrillary acidic protein (GFAP) was used to portray the morphology of astrocytes. The hypoxia-inducible factor 1 (HIF-1) staining was performed to confirm hypoxia induced cell death and there was a dramatic expression of HIF-1α displayed in exposed astrocyte cells compared to the control. In molecular level, genes were chosen, such as glyceraldehyde 3-phosphate dehydrogenase (GAPDH), GFAP, HIF-1α and B-cell lymphoma 2 (Bcl-2) and ran the reverse transcription-polymerase chain reaction (RT-PCR).

**Results:**

Microscope revealed a filamentous and clear nucleus appearance in a control whereas the rupture nuclei with no rigid structure of the cell were found in the 3% oxygen. The control and hypoxia cells were also stained with the annexin V-fluorescein isothiocyanate (annexin V-FITC). Fluorescence microscope reveals astrocyte cells after hypoxia showed higher expression of nuclei but not in control. Merging PI and FITC showed the differences of nuclei expression between the control and hypoxia. In the molecular analysis, there were significant changes of GFAP, HIF-1α and Bcl-2 in hypoxia exposed cells when compared to the control group.

**Conclusion:**

Cells that were exposed to hypoxia (3% oxygen for 15 min) clearly showed damage. General view of human hippocampal astrocyte genomic response to hypoxia was obtained.

## Introduction

In the human brain, the hippocampus is considered one of the most highly sensitive regions to hypoxia. Exposure to hypoxia triggers several disastrous effects on the central nervous system, especially on the neurological and physiological aspects. Reduction of oxygen may also alter the brain’s structure (1, 2). At the molecular level, hypoxia may lead to an increase in free radical generation, oxidative stress, L-type calcium channels and cholinergic dysfunction (3–5). Moreover, hypoxia exposure to the hippocampus may cause apoptosis or necrosis, which leads to impaired learning and memory (6). There is also growing evidence in the literature that clearly shows the effect of oxygen alteration on the brain, which may lead to neurodegenerative disorders and impaired cognitive function (7, 8).

The activation of astrocyte cells, also known as astrogliosis, happens as part of the response of the central nervous system (CNS) to critical situations, such neurotrauma, brain injury, ischaemic damage and neurodegenerative diseases (9, 10). Regardless of its origin, the hallmark of reactive astrocyte is an increase not only of glial fibrillary acidic protein (GFAP) expression but also of vimentin and nestin. Generally, GFAP, vimentin and nestin can be categorised as building blocks of intermediate filaments (IFs), which form the cytoskeleton along with microtubules and actin filaments (11). However, there are several visible distinctions between rodent and human astrocytes. One of the differences is the classes of GFAP expression. Compared to rodents, human protoplasmic astrocytes are larger and more complex than rodent cells (12).

To mimic the effects of hypoxia, several methods have been used. Some metals, such as cobalt chloride, nickel chloride and deferoxamine (13), have long been used as hypoxia mimicking agents. The advantages of using these chemical agents in mimicking hypoxia condition are because they are inexpensive and fast. Although they mimic hypoxia by inducing hypoxiainducible factor 1 (HIF-1), they also may interfere with other cell’s genes. For this research, a human hippocampal astrocyte cell was used and was exposed to different oxygen concentration for 15 min. In mimicking hypoxia, a chamber with adjustable gases was used. Hypoxic chambers have the advantage of not using drugs that can alternate cell behaviour independently of the oxygen tension. However, not all types of experimentation can be done, as oxygen reenters the chamber at each opening and thus lessens hypoxia.

In the CNS, astrocytes are the most abundant cells and they play vital roles for the neuron networks to function efficiently. Although neurons and neighbouring astrocytic glial cells have been studied widely in nervous tissue from animals, mostly from rats or mice, their properties and interactions in the human brain are less known. Characterisation and properties of astrocytes in the CNS are basically derived from studies on rodent brains. However, few studies have used human astrocytes, which have shown some unique anatomical features that are absent in a rodent’s CNS (14). Moreover, no studies have focused on the effect of human astrocytes in any critical condition. For this research, we used human cell models that mimic the CNS, a human hippocampal astrocyte cell line, and we then exposed the cells to a critical condition, specifically hypoxia. Generally, we wanted to gain a better understanding of the human CNS and how it responds to oxygen deprivation. After exposure, a cell viability assay using trypan blue was performed.

Then, to better understand cell characterisation before and after hypoxia exposure, several reliable immunofluorescent assays, using GFAP, hypoxia-inducible factor 1 alfa (HIF-1α) and annexin V-fluorescein isothiocyanate **(**FITC) apoptosis detection, were performed. As the molecular basis of these cells and their sensitivity towards this kind of situation remain unclear, this study is a great opportunity to deeply investigate the characterisation of astrocyte cells before and after hypoxia exposure. Thus, we also performed a molecular analysis by running the reverse transcription-polymerase chain reaction (RTP-CR) for several genes. We hypothesise that different levels of oxygen concentration may impact differently on the human hippocampal astrocyte cells in vitro on the morphological molecular levels.

## Methods

### Study Design

This was a lab-based exploratory in vitro study on human hippocampal astrocyte cell culture. The research was carried out at the Neuroscience Laboratory in the Department of Neurosciences and Central Research Laboratory, School of Medical Sciences, Universiti Sains Malaysia. A total of five groups of astrocytes cells including the control group, were used in this study for the research experiment. The cells were then exposed to different oxygen concentrations; 15% O_2_, 10% O_2_, 5% O_2_ and 3% O_2_ in 15 min.

### Culture Procedures and Conditions

All cell culture methods were performed inside a Biosafety Cabinet Class II biohazard safety cabinet (Nu-437-400E, Nuaire, Plymouth USA) and under stringent aseptic conditions to avoid contamination. Good cell practice (GCPP) guidelines (15) were practiced throughout the research project. The human hippocampal astrocyte cell line (HA-h, Cat.#1830) which was procured from ScienCell line, Carlsbad, CA, USA was used in this study. Cat.#1830, HA-h from ScienCell Research laboratories is characterised by immunofluorescence with antibodies specific to GFAP. HA-h is negative for HIV-1, HBV, HCV, mycoplasma, bacteria, yeast and fungi. These cells were isolated from the human hippocampus and were cryopreserved at passage; one in each vial contained > 5 × 105 cells in 1 mL volume. The cells were cultivated in cell culture flask 25 cm^2^ filter cap, T25 (BA6F271, SPL Lifescience, Gyeonggi-Do, Korea) in a normal atmosphere of 5% CO_2_ and 95% air (Linde Malaysia Sdn. Bhd., Malaysia) with regulator at 37 °C incubator (Heracus, Germany). These human hippocampal astrocytes cells were grown in specific astrocyte media (AM, Cat.#1801, ScienCell, Carlsbad CA, USA) containing astrocyte growth supplements (AGS, Cat.#1852, ScienCell, Carlsbad CA, USA), foetal bovine serum (FBS, Cat.#0010, ScienCell, Carlsbad CA, USA) and penicillin/streptomycin solution (Cat.#0503, ScienCell, Carlsbad CA, USA). The growth media was prepared by adding 500 μL AGS, 1 mL of FBS and 500 μL penicillin/streptomycin to 50 mL of media in a sterile 50 mL falcon tube. Prepared complete media was kept at 4 °C (XMA, Malaysia). The cryovial containing frozen stock was retrieved from liquid nitrogen stored in the Department of Pathology, Health Campus USM and thawed in a 37 °C water bath (Memmert, WNE 7, Germany). The thawed cells were suspended before being dispensed into cell culture vessels, T-25 flasks (SPL Lifescience, Gyeonggi-Do, Korea) containing pre-warmed media. The cultured flask was incubated in a humidified atmosphere CO_2_ incubator (Heracus, Germany) with 5% CO_2_ (Linde Malaysia Sdn. Bhd., Malaysia) at 37 °C overnight to allow cell attachment. The culture medium was refreshed the next day to remove residual DMSO (Sigma Aldrich, Saint Louis MO, USA) and unattached cells. All the experiments were carried out using passage one.

### Hypoxia Exposure

In mimicking the hypoxia condition, a chamber (Model C374, BioSpherix, Union St Parish, NY USA) with adjustable mixture of oxygen and carbon dioxide gasses (Linde Malaysia Sdn. Bhd., Malaysia) was used. The astrocyte cells were plated on 12-well culture plates (Cat no: 0030721012, Eppendorf AG, Harmburg, Germany) with a 15 mm cover slip (EMS, Pennsylvania USA) and then were exposed to hypoxic condition via hypoxia chamber. At first, the cells were exposed to hypoxia at various time points; 5 min, 10 min, 15 min, 20 min, 25 min and 30 min at hypoxia, 3% O_2_. Then, 15 min was chosen as the time point. The cells were then exposed to different oxygen concentrations; 15% O_2_, 10% O_2_, 5% O_2_ and 3% O_2_ in 15 min (16, 17).

### Cell Viability Assay Using Trypan Blue Dye Exclusion

After exposing cells to different hypoxic conditions, cells are harvested with Trypsin/EDTA (T/E 0.25%, Cat.#0103, ScienCell, Carlsbad CA, USA) by incubating them for about 1 min. After transferring detached cells into a 15 mL centrifuge tube (Costar ®, Corning Incorporated, USA), the cells were centrifuged at 3,250 rpm (Benchtop centrifuge, Universal 320R, Hettich Tuttlingen, Germany) for 3 min at 4 °C and pellets of the cells were collected. Then, the cells were mixed with 0.5 mL of 0.4% Trypan blue dye solution (Cat.#0203, ScienCell, Carlsbad CA, USA). After 5 min–10 min, the cell suspension-Trypan blue mixture was transferred to both chambers of the haematocytometer (Marienfed, Germany). Then, the percentage of cell viability was calculated by an automated countess (CountessTM automated cell counter Model C10227, Invitrogen, Carlsbad, CA USA) (18, 19).

### Glial Fibrillary Acidic Protein Immunofluorescence Assay

A GFAP immunofluorescence assay was performed to ensure the architecture and morphology of astrocyte cells. After hypoxia exposure, each group of astrocyte cells were fixed with 4% paraformaldehyde (Fisher Scientific, New Jersey USA) to preserve the cellular morphology. Then, the cells were washed with the phosphate bukffer saline (PBS) (ScienCell, Carlsbad CA, USA) three times and were permeabilised with 0.1% Triton X-100 (Triton® X-100, Sigma Aldrich, Saint Louis MO, USA) for about 20 min. One percent of BSA/PBS was placed on the cells for 45 min. Blocking with 1% bovine serum albumin (BSA)/PBS is an important step in minimising unspecific binding of the primary antibody within the cell. After sample preparation by fixation, permeabilisation and blocking, the actual immunoreaction occurs. The cells were incubated with the specific primary antibodies (1:200), PierceTM GFAP antibody (#PA5-18598, Thermo Scientific, Rockford IL, USA) overnight at 4 °C to mark the desired target structures. Subsequently, a crucial washing was needed after incubation with the GFAP to reduce nonspecific binding of the secondary antibody. The cells were then incubated with secondary antibody (1:400), rabbit anti-goat IgG (H+L) secondary antibody, FITC conjugate (#31509, Thermo Scientific, Rockford IL, USA) for about 1 h, diluted with blocking solution. This incubation step was done in darkness to prevent fluorochrome bleaching. The coverslip 15 mm (EMS, Pennsylvania USA) with the cells laid upside down onto the microscope slide contained a drop of mounting medium. The cells were ready to be viewed under fluorescence microscope with an image analyser (Olympus BX14 32P02, Olympus, Tokyo Japan) (20).

### Annexin V-FITC/Propidium Iodide Staining

The apoptosis detection was performed using TACSTM annexin V-FITC apoptosis detection kit TREVIGEN (Cat. no TA4638, Trevigen, Gaithersburg, Maryland USA). After being exposed to different oxygen levels, coverslips from each group were washed with PBS to remove any debris. Then, a 100 μL annexin V incubation reagent was prepared of approximately for each group. The annexin V incubation reagent was placed onto the samples and was gently spread to ensure the samples were covered. The coverslips with cells were incubated with the reagent for about 15 min at room temperature in a dark room. Afterwards, the coverslips were washed with a 50 mL 1× binding buffer twice for 2 min. Drops of fluorescence compatible mounting medium, Dako Fluorescent Mounting Medium (Dako North America, CA, USA) were placed on the slides and the cells were ready to be viewed under Fluorescence Microscope with image analyser (Olympus BX14 32P02, Olympus, Tokyo Japan) (21).

### Hypoxia-Inducible Factor 1α Staining

As HIF-1 is expressed in most cell types in response to hypoxia (22), HIF staining was performed in control and exposed astrocytes cells to point out the difference in HIF expression among five groups. After following hypoxic conditions at different oxygen levels, each cell from each group was fixed with 4% paraformaldehyde (PFA) (Fisher Scientific, New Jersey USA). Then, after being washed with PBS, they were permeabilised with 0.1% Triton X-100 (Triton® X-100, Sigma Aldrich, Saint Louis MO, USA)/PBS for about 15 min to 20 min. Again, the cells were washed with the PBS and then were blocked with 1% BSA/PBS for 45 min. Immediately after washing, the cells were incubated with the primary antibody, HIF-1α mouse monoclonal IgG (#2513, Thermo Scientific, Rockford IL, USA) overnight. Afterwards, the cells were washed again to ensure well binding. The cells were then incubated for 1 h with rabbit anti-mouse IgG secondary antibody FITC conjugate (#31509, Thermo Scientific, Rockford IL, USA). After washing and placing the mounting media, Dako fluorescent mounting medium (Dako North America, CA USA) onto the slide, the coverslips with cells were ready to be viewed under the fluorescence microscope with image analyser (Olympus BX14 32P02, Olympus, Tokyo Japan) (23).

### Reverse Transcription-Polymerase Chain Reaction

For the molecular analysis, we were using TRIzol® RNA Purification kits combined with PureLink™ RNA® mini kit (Kits cat. no. 12138555, Thermo Scientific, Rockford IL, USA) (24). The RNA isolation was done in three groups: i) control; ii) hypoxia (15% O2) and iii) hypoxia (3% O2). After exposure to the hypoxic, the cells in the flask were harvested by using trypsin/EDTA (T/E 0.25%, Cat.#0103, ScienCell, Carlsbad CA, USA) then, the pellets of the cells were gained by centrifuge (Centrifuge 5417R, Eppendorf, Harmburg Germany) the cells at 3250 rpm for 3 min at 4 °C. Then, the pellets were submerged in PBS (Sigma Aldrich, Saint Louis MO, USA) and ready for the isolation work. After removing the PBS, 500 μL of TRIZOL agents (Kits cat. no. 12138555, Thermo Scientific, Rockford IL, USA) were added to the pellet and were incubated 5 min at room temperature to homogenise them and allow complete dissociation. Then, 200 μL of chloroform (Kits cat. no. 12138555, Thermo Scientific, Rockford IL, USA) were added and was vortexed vigorously for about 15 s. Later, the samples were centrifuged at 12,000 × g for 15 min at 4 °C. Following centrifugation, the mixtures were separated into a lower red, phenol-chloroform phase, an interphase and a colourless upper aqueous phase. The RNA of cells remains in the aqueous phase. To complete the phase separation, the upper aqueous phase was transferred to the new tube (SPL Lifescience, Gyeonggi-Do, Korea) cautiously without disturbing the interphase.

RNA from the aqueous phase was extracted by mixing the sample with isopropyl alcohol (Kits cat. no. 12138555, Thermo Scientific, Rockford IL, USA). Five hundred microlitre of isopropyl alcohol was needed in each reaction with 1 mL of TRIzol. Following incubation of samples at 15 °C–30 °C for 10 min and centrifuge at 12,000 × g for 10 min at 4 °C (Centrifuge 5417R, Eppendorf, Harmburg Germany), the RNA precipitation was visible. Then, 500 μL of 70% alcohol was used in the washing phase after the supernatants were removed completely. This washing phase was repeated once. Afterwards, all leftover ethanol were discarded and the RNA pellets were air dry for about 5 min. For the redissolving RNA, 20 μL DEPC treated water (Kits cat. no. 12138555, Thermo Scientific, Rockford IL, USA) was used onto the RNA samples. The sample concentration and purity were checked by a NanoDropTM 2000 spectrophotometer (ThermoFisher Scientific, Waltham MA, USA). The A260/A280 ratio should be above 1.6 (25).

For the cDNA synthesis reaction, a kit of SuperScript IV first strand synthesis system (Kit Cat. no 18091050, Thermo Scientific, Rockford IL, USA) was used (24, 26). The first step of this reverse transcription reaction was to combine all components in the PCR reaction tube (SPL Lifescience, Gyeonggi-Do, Korea). The total up to 13 μL of the mixture consisted of 50 μM Oligo d(T), 10 mM dNTP mix, RNA templates and DEPC-treated water (Kit Cat. no. 18091050, Thermo Scientific, Rockford IL, USA). All components were mixed well by centrifuge together (Centrifuge 5417R, Eppendorf, Harmburg, Germany). Then, the RNA-primer mixes were heated at 65 °C for 5 min and afterwards were incubated for about 1 min on ice (Molecular Department, USM). Then, RT reaction mix water (Kit Cat. no 18091050, Thermo Scientific, Rockford IL, USA) that consists of a 5x SSIV buffer, 100 mM DIT, ribonuclease inhibitoe and SuperScript IV reverse transcriptase were mixed together. RNA-primer mixes, the annealed RNA was then combined together with the RT reaction mix and were incubated at 23 °C for 10 min. The following primers were used. After that, the combined reaction mixtures were incubated at 55 °C for 10 min and then were incubated again at 80 °C for 10 min to inactivate the reaction. To remove RNA from the mixture, 1 μL E. coli RNase H was added and then incubated for 20 min at 37 °C (24, 26). The following primers has been used: GAPDH (206 bp): FP: 5′-AGG GCT GCT TTT AAC TCT GGT -3′RP: 5′-CCC CAC TTG ATT TTG GAC GGA -3′HIF (649bp): FP 5′-GGC GCG AAC GAC AAG AAA AA -3′RP: 5′-GCA CCA AGC AGG TCA TAG GT -3′GFAP (465bp): FP: 5′-GCA CGC AGT ATG AGG CAA TG -3′ RP: 5′-GGC TGG TTT CTC GAA TCT GC -3′ Bcl-2 (513bp): FP: 5′-TGG ACG GAG TAG CTC CAA GA -3′RP: 5′-CCA CCC CAG GAT CTA ACA GC -3′.

The protocol for PCR amplification is based on the protocol of Platinum® PCR SuperMix (Kit cat. no. 11306016, Thermo Scientific, Rockford IL, USA). Qualified reagents were provided in this PCR SuperMix in order to amplify the nucleic acid templates by PCR. The PCR reaction, it was recommended to assemble on ice to ensure the quality of the results later. For each reaction vessel, several components are needed, including 45 μL platinum, 1 μL primers and 1.5 μL template DNA solution (Kit cat. no. 11306016, Thermo Scientific, Rockford IL, USA). The reaction vessels were capped and were loaded into a thermal cycle at 94 °C for about 2 min. This process is essential to ensure the templates are denatured completely and the enzymes are activated. Afterwards, 35 cycles of PCR amplification were performed at specific temperature and timing which was provided in the protocol. PCR amplification was started by the denature process at 94 °C for 30 s, following the annealing process at 59 °C for 30 s and lastly, the extended process at 72 °C for 1 min per kb. The denature, anneal and extend process was done in a thermal cycler (ThermoFisher Scientific, Waltham MA, USA) (3, 27).

### Statistical Analysis

SPSS version 20 from 16 June 2018 IBM, USA, was used for all statistical analysis. All values were expressed in mean ± SD. Comparisons between groups were performed using one-way analysis of variance (ANOVA) followed by post-hoc analysis with Tukey’s correction method.

*P-value of < 0.05 is considered significant.

## Results

### Cell Culture Characteristics and Morphology

Cell culture was initiated using the human hippocampal astrocyte cell line. The cell was cultivated on a T-25 flask and was incubated for 24 h to ensure the cells were attached. Cells are then maintained in a specific medium containing the desired amount of AGS, FBS and penicillin/streptomycin solution. Within 48 h, cells began to clump together in tightly packed aggregates. Cells within aggregates would then begin to proliferate and increase in size and number. After several days, more processes developed and elongated to form connections with neighbouring cells. Cell cultures were observed for several weeks as astrocyte cells began to confluent and were ready to be used for the experiment.

### Trypan Blue Dye Exclusion for Cell Viability

**Figure 1:**
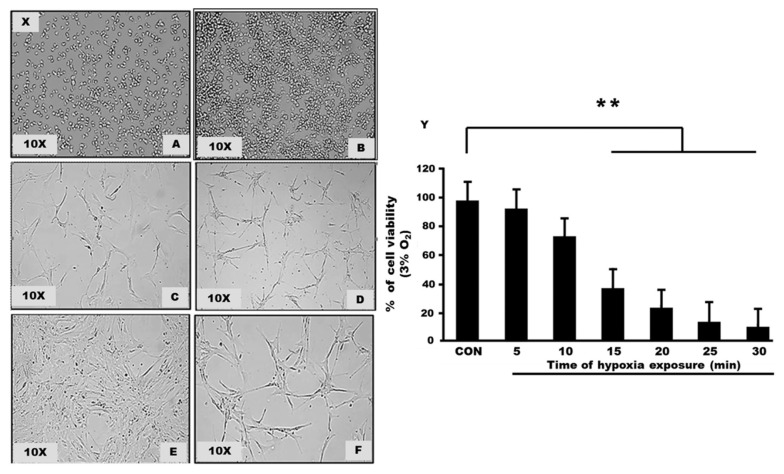
**(X):** Culturing human hippocampal astrocyte cells line in astrocyte media containing essential supplements for cells to growth (Light Microscope Zeiss, 10×). (A) Day 1: Initiated culture from 1 mL of vial containing > 5 × 10^5^ cells/mL. (B) Day 3: Cells start to accumulate and clump together. (C and D) After several days, processes of cells start to form and continue to develop more branches (E) Configuration of star-like cells start to appear after 10–14 days. Cells accumulate and reach more than 90% confidence. (F) After subculture and reach 70%–90% confluent, the cells were ready for experiment; passage 2 and passage 3 cells were used in this research. **(Y):** The cell viability of astrocyte cells following exposure to hypoxia (3% Oxygen). Graphs show the mean of cell viability for each group including the unexposed cell (0 min); control group. There are significant changes between control and hypoxia exposed groups (*P*-value < 0.001)

A pre-test was performed to define cell survival in a hypoxic condition. We exposed the astrocyte cells to hypoxia, 3% of oxygen percentage at different time points; 0 min, 5 min, 10 min, 15 min, 20 min, 25 min and 30 min. Then, we accessed the cell’s viability by Trypan blue dye exclusion. After that, based on the pre-test result, we choose 15 min as the time point and expose astrocyte cells to various oxygen levels. Five groups, including the control, were categorised based on different oxygen percentages: 15% oxygen, 10% oxygen, 5% oxygen and 3% oxygen (Figure 1).

### Apoptosis Detection via Annexin V-FITC Staining

**Figure 2:**
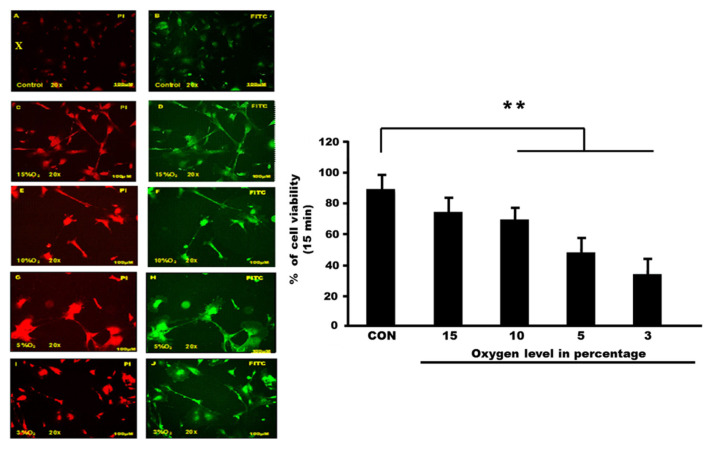
**(X):** PI and FITC staining of human hippocampal astrocyte cells (Filter wavelength used; WU = 330 nm–385 nm/WB = 460 nm–490 nm). Under fluorescence scanning, PI displayed a red image while in FITC the cells were displayed in green. (A) and (B) control group, (C) and (D) astrocytes were exposed to 15% of oxygen, (E) and (F) cells exposed to 10% of oxygen, (G) and (H) cells exposed to 5% oxygen and (I) and (J) astrocytes cells exposed to 3% oxygen. **(Y):** The cell viability of astrocyte cells following exposure to different percentages of oxygen level at a constant time point. Astrocyte cells were exposed at different oxygen percentages; 15% oxygen, 10% oxygen, 5% oxygen and 3% oxygen in 15 min. Graphs show the mean of cell viability for each group including the unexposed cell (control group). There are significant changes between control and exposed groups (*P* < 0.005)

**Figure 3:**
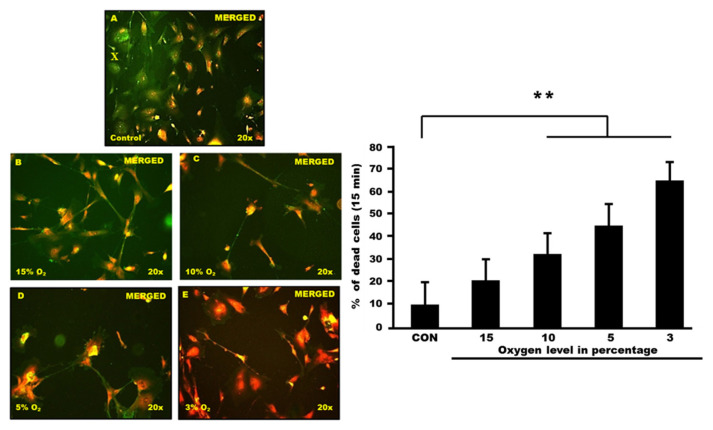
**(X):** Merged between PI and FITC clearly showed the differences of nuclei expression between the control and hypoxia exposed group. The expression intensity was observed from (A) control, (B) astrocytes were exposed to 15% of oxygen, (C) cells exposed to 10% of oxygen, (D) cells exposed to 5% oxygen and (E) astrocytes cells exposed to 3% oxygen. All images were counted randomly by 3 blinded investigators (Cronbach’s alpha: 0.965). (Filter wavelength used; WU = 330 nm–385 nm/WB = 460 nm–490 nm). **(Y):** The cell death (FITC) of astrocyte cells following exposure to different percentages of oxygen level at constant time point. Astrocyte cells were exposed at different oxygen percentages; 15% oxygen, 10% oxygen, 5% oxygen and 3% oxygen in 15 min. Graphs show the mean of cell death for each group including the unexposed cell (control group). There were significant changes between control and exposed groups (*P* < 0.001)

Control and hypoxia cells were stained with the annexin V-FITC and then observed under a fluorescence microscope. Cells with high fluorescence intensity due to chromatin condensation or nuclear fragmentation are considered as apoptotic cells. The number of high-fluorescent condensed or fragmented nuclei was counted randomly in each group. Results are expressed as the percentage of condensed or fragmented nuclei relative to the total number of cells counted for each experimental condition (Figures 2 and 3).

### Portray Astrocyte Structure by GFAP Staining

**Figure 4:**
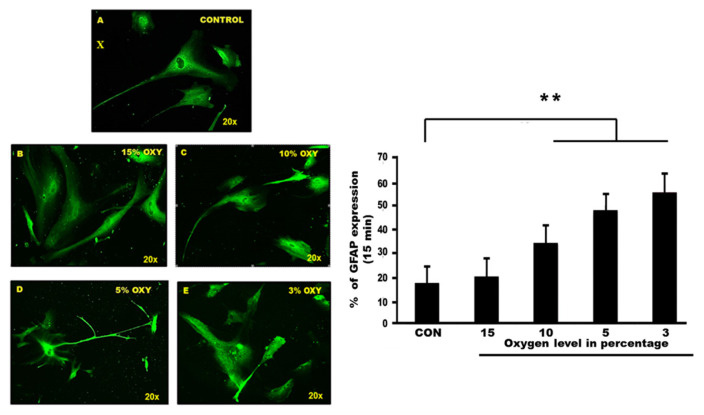
**(X):** Staining human hippocampal astrocyte cell line using the GFAP (primary antibody) and anti-goat (secondary antibody). Fluorescence scanning microscope revealed a filamentous and clear nuclear appearance in a (A) control group. Different intensity can be found in (B) cells exposed to 15% oxygen, (C) cells exposed to 10% oxygen and (D) cells exposed to 5% oxygen. The rupture nuclei along with no rigid structure of the cell were displayed in (E) hypoxia group, the 3% oxygen exposure. **(Y):** The percentage of astrocyte cells in different oxygen concentrations correspond to GFAP. There were five groups of astrocyte cells exposed at different oxygen percentages; 15% oxygen, 10% oxygen, 5% oxygen and 3% oxygen in 15 min. Graphs show the mean of cell nuclei with positive GFAP for each group. One way Anova showed significant changes between control and exposed groups (*P* < 0.001)

GFAP is considered as one of the reliable markers for characterising astrocyte cell structure. In this study, GFAP acts as a primary antibody conjugated with anti-goat-FITC as a secondary antibody. The GFAP marked cells and displayed the structure and nuclei of astrocyte cells. The number of cell nuclei corresponding to GFAP (+) astrocytes was counted. For such cell counting, five microscopic images were randomly chosen. One-way ANOVA showed *P*-value = < 0.001 with Cronbach’s alpha = 0.963, indicating high internal consistency (Figure 4).

### Hypoxia-Inducible Factor Staining in Confirming the Death of Astrocyte Cells

**Figure 5:**
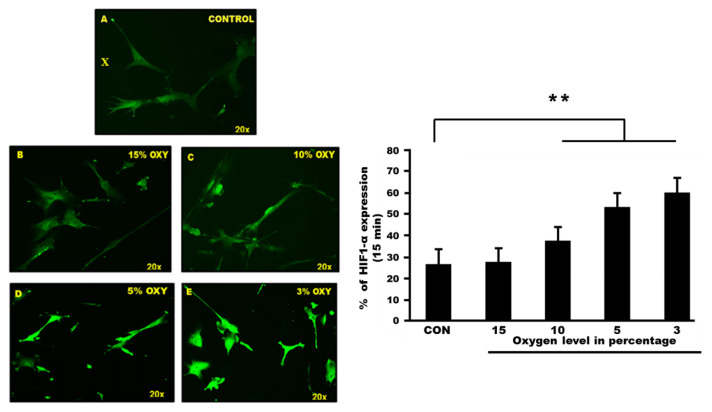
**(X):** HIF staining human hippocampal astrocyte cell line. The green image stained cell showed the reactive astrocyte. (A) Control group showed poor intensity of HIF staining. Different intensity of HIF can be found in (B) cells exposed to 15% oxygen, (C) cells exposed to 10% oxygen. The intensity of the expression was higher in (D) cells exposed to 5% oxygen and (E) cells exposed to 3% oxygen. **(Y):** The percentage of astrocyte cells in different oxygen concentration and its HIF-1α expression. Five groups of astrocyte cells exposed at different oxygen percentages; 15% oxygen, 10% oxygen, 5% oxygen and 3% oxygen for 15 min showed different expressions of HIF-1α. Statistical analysis using one way Anova showed significant changes between control and exposed groups, *P* < 0.001 with Cronbach’s alpha: 0.950

In determining the regulation of HIF-1 in hypoxia, we compared HIF-1α expression between control and exposed cells. The HIF-1 staining was also performed to confirm the cell death due to hypoxia exposure. Based on the fluorescence microscope view, there are dramatic expressions of HIF-1 that were displayed in exposed astrocyte cells compared to the control. The scanning fluorescence result also showed different image intensity of astrocyte cells after exposure to different oxygen concentrations. Random counting was performed by three blinded investigators (Figure 5).

### Molecular Analysis of Astrocyte Cells for Control and Hypoxia

**Figure 6:**
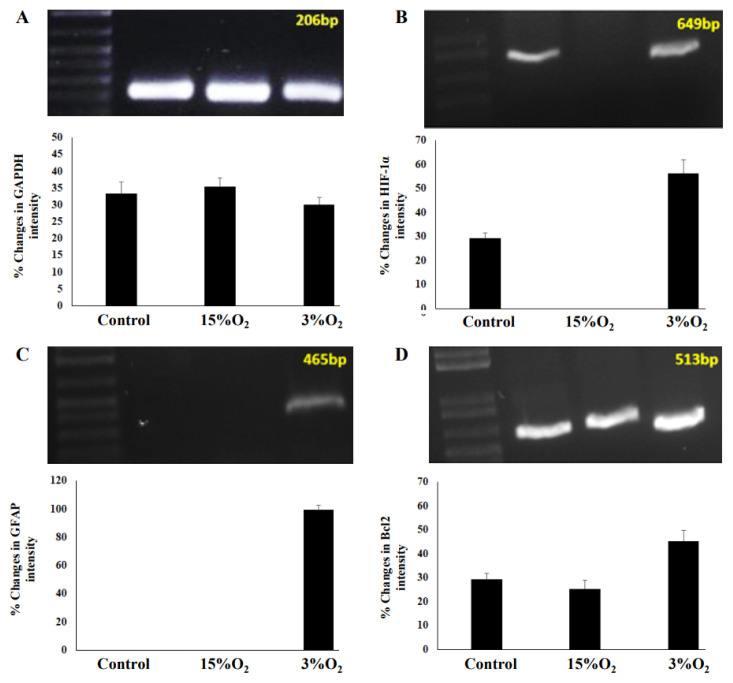
Molecular analysis involving GAPDH, GFAP, HIF-1α and Bcl-2 in control and hypoxia group. Different expressions and different percentages changes in intensity were displayed in (A) GAPDH, (B) GFAP, (C) HIF-1α and (D) Bc1-2

For better understanding of gene expression induced by hypoxia, we ran a molecular analysis involving RT-PCR and electrophoresis. By understanding changes in gene expression of human hippocampal astrocytes following hypoxia exposure, it could reveal new mechanisms of brain tolerance. Based on the result, expressions of four genes (GAPDH, GFAP, HIF-1α and Bcl-2) in each group were regulated differently (Figure 6).

## Discussion

While significant effort has gone into studying the astrocyte cells derived from rats and mice, no studies have examined human hippocampal astrocyte cell culture. Thus, much of this project was devoted to developing and optimising an effective protocol to culture astrocyte cells from the human hippocampus. This study demonstrated a successful human hippocampal astrocyte cell culture in vitro. Difficulty in keeping the cells kept alive and growth were anticipated, but the culture was successfully done. The use of specific media along with astrocyte growth supplements and streptomycin/penicillin solution has been shown to be an important element for the survival and growth astrocyte cultures. Overall, the astrocyte cell culture protocol developed as part of this study allows for effectively studying the characterisation of human hippocampal astrocyte cells following hypoxia exposure.

One of the essential parameters for this study was the viability of cultured human hippocampal astrocyte cells. From the result gained in the pre-test, we found that cell viability was reduced as the timing of hypoxia exposure increased. This was significant in previous studies. Zhu et al. (28), for example, showed that the viability of Schwann cells was reduced after exposing the cells to hypoxia at different points in time. Our results also showed there was significant difference between cell viability in control and exposed cells to different oxygen concentrations. Exposure of cultured human hippocampal astrocytes to hypoxia—that is, 3% oxygen within 15 min—prompted about 70% of the cells to die. The result was correlated to a previous study, as Trajano et al. (29) found that cell viability of cultured myoblast cells decreased as the FBS concentration and oxygen level decreased. They also found that even though the level of the reactive oxygen species was not altered, but the changes of the cells’ physiologic conditions affected the cell viability, apoptosis and necrosis in myoblast cells. This result indicates that human hippocampal astrocyte cells decrease as the oxygen concentration decreases. Further investigation was performed in this study to clearly understand the mechanism of astrocyte cells.

Numerous culture studies demonstrated apoptosis and other forms of programmed cell death in astrocyte cells, related to different events, such as ischemia, acidosis, oxidative stress, substrate deprivation and cytokinesis (30, 31). In detecting the degree of human hippocampal astrocyte cell death, annexin V-FITC apoptosis kit was used. Annexin, a Ca^2+^ dependent phospholipid-binding protein, has higher affinity to membrane phospholipid phosphatidylserine (PS). PS translocation results in the loss of cell membrane loss its integrity, which follows the stages of cell death, either apoptosis or necrosis (21).

A general conclusion from our current findings was that human hippocampal cells line are sensitive to a reduction in oxygen due to hypoxia. The progression of cell death was differentiated based on the degree of annexin V and PI staining. In tracking cell death progression, cells usually can be tracked from annexin V and PI; when they are both negative, the cells are viable. However, when annexin Vi is positive and PI is negative, it indicates early apoptosis. For the late stage of apoptosis, there are double positives of annexin V and PI (32, 33). Our results showed that weak annexin V staining was observed only on the cellular membrane of live cells, while the apoptotic cells showed higher degrees of cell surface labelling. Our results corresponded with previous studies that found that observed membrane stains by annexin V and strong nuclear staining from PI in dead cells (34, 35). Other studies demonstrated that cells in the early apoptotic stage showed a green staining with annexin V-FITC but did not show any strong yellow-orange PI fluorescence (21, 36). Even though this staining does not directly stain DNA, it does help in identifying events that alter the DNA and initiate cell death (37). Our data further suggest that in combination with vital dye staining, annexin V-FITC binding assays can be used to monitor the progressive stages of apoptosis.

To identify the cell structure of human hippocampal astrocytes, we used GFAP as a marker, as it is useful to identify astrocytes in a highly specific manner. Our findings suggested that GFAP stained in control and exposed cells were displayed differently, indicating that there were changes of structural patterns following hypoxia exposure. Statistically, our data showed that there were significant differences among the groups. The GFAP marker pictured a well-shaped cell with full GFAP-immunoreactive in the control group, while in the hypoxia exposed group, the GFAP staining demonstrated that the area of the section occupied by GFAP-immunoreactive processes and cell bodies had significantly decreased. In the hypoxia exposed group, (3% oxygen exposure for 15 min), the structure of the cell bodies diminished, as they had no clear nucleus pattern. This result was corroborated by previous studies. In an animal study related to white matter astrocytes and hypoxic/ischemic neonatal pig brains, the authors used GFAP as a marker and compared the complete architecture of white matter astrocytes in control and hypoxic/ischaemic brains (38). They found that individual astrocytes in the control white matter group had multiple long, branching processes expressing GFAP, while the white matter astrocytes from the H/I brain appeared to have a reduced expression of GFAP, with only a few shorter processes expressing GFAP. Other studies found that GFAP-positive reactive astrocytes were significantly increased in the cortical regions of the human ischemic brain, compared to adjacent normal tissues and the control subjects (39). Our findings suggested that the exposure cultured human hippocampal astrocyte cells to hypoxia for 15 min strongly up-regulated the GFAP, which is the hallmark of astrogliosis responding to the injury.

An important factor in hypoxia is HIF-1. HIF-1 is considered the master regulator of hypoxia-responsive genes and much evidence shows its induction in adaptations to low oxygen conditions, especially in brain ischaemia (40, 41). Various genes, including those involved in angiogenesis or oxygen transportation, are regulated by HIF-1 during hypoxia. In mammalian cells specifically, HIF-1 activation is a hallmark of hypoxia. Due to its importance, it is commonly used in hypoxia screening (42, 22).

In this study, we applied HIF staining to the control group and the different groups of hypoxia exposed cells to confirm that the damage or cell death was caused by oxygen reduction. Our result showed that there were up-regulations of HIF-1 staining expression. Compared to exposed cells, unexposed cells demonstrated a poor expression of HIF-1. This analysis was relayed to previous data, as no immunoreactivity of HIF-1α staining was observed in the sham groups of rats, while massive HIF-1α immunoreactivity was detected in the infarction region (43). Meanwhile, in a study related to aging, where there were changes of cerebral blood flow, the authors found that there were increases of HIF-1α expression, suggesting that aging promoted HIF-1α expression (44). Although there was no remarkable HIF-1 expression of human hippocampal astrocyte cell lines in previous studies, some studies did report that there were inductions of HIF-1α expression in several cell lines after exposure to the hypoxic/ischaemic condition (45). In order to obtain a general view of the human hippocampal astrocyte genomic response to hypoxia, molecular analysis was performed in this study. Several genes, including GAPDH, GFAP, HIF-1α and Bcl-2, were chosen and ran using RT-PCR. Based on our results, the expression of the GAPDH gene seemed to be constant in all groups, even with no significant differences in the control group. The slight differences of the GAPDH gene were due to its roles as a housekeeping gene as well as a control gene. It is also commonly used as an internal standard in monitoring loading variations in gene analysis. The gene was found to play an independent role in cellular maintenance and its expression (46, 47). Increased GFAP expression is considered an indicator for the reactive astrocyte (48). A previous study found that there were elevated levels of GFAP expression in astrocyte cells after the ammonia exposure but not in the control group (49). Consistent with this, we found that there were up-regulated GFAP expressions in the hypoxia group but no significant differences between the hypoxia and control groups. Our findings indicated that any critical situation that disturbs the brain environment will lead to reactive astrocytes and an increase in GFAP expression.

HIF-1α changes are commonly mentioned in hypoxia studies, as its expression is tightly regulated by cellular oxygen concentration (50). Basically, this gene acts as a determinant for HIF-1α DNA binding activity during hypoxia. As shown in our results, exposure of human hippocampal astrocyte cells to hypoxia (3% oxygen) induced HIF-1α expression slightly expressed in control but not in hypoxia (15% oxygen). Our findings suggested that HIF-1α is more inducible by hypoxia exposure in human hippocampal astrocytes compared to hypoxia exposure, suggesting that HIF-1α could play role in mediating the precondition effects of hypoxia (51). Bcl-2 is categorised as part of the B-cell lymphoma-2 family, which regulates apoptosis activities, mainly at the mitochondrial level. The increased expression of this protein indicates the appearance of an apoptosis event (52, 53). However, in the present study, Bcl-2 showed significant expression in 3% oxygen, although other groups expressed Bcl-2. Bcl-2 also was expressed in control groups as well, which may be due to activation of the cellular pathway to maintain the genomic stability. Our results also showed a highly up-regulated Bcl-2 level in human hippocampal astrocyte cells, indicating there was regulation of cell death and autophagy (52). The increase of Bcl-2 expression in all groups, however, is not sufficient to understand the genomic apoptotic behaviour of human hippocampal astrocyte cells. It is, however, interesting to note that the elevation of this protein in hypoxia groups was partly due to hypoxia exposure (53).

We conclude that human hippocampal astrocytes underwent morphological changes as well as molecular changes differently based on their exposure to different levels of oxygen concentration. The structural changes of astrocytes can be seen clearly when we compare between the control group and the group of exposed cells. Cells that were exposed to hypoxia (3% oxygen for 15 min) clearly showed damage, as their nuclei ruptured and they lost their structural rigidity. In the molecular analysis, there were significant changes of GFAP and HIF-1α in hypoxia exposed cells when compared to the control group. Thus, we could conclude that morphological astrocyte cells begin to change after hypoxia exposure. Future studies of human hippocampal astrocyte cells with more reliable markers and genes along with advanced methods are necessary to obtain more information on astrocyte mechanisms. Another key question is whether astrocytes have higher resistance compared to neurons and whether astrocytes in the CNS could survive in hypoxic environment. Regardless of this study’s weaknesses, the findings in this research may become a starting point in enhancing our understanding about the potential of astrocytes and how they may react in critical situations.
